# Uncovering Inherited Cardiomyopathy With Human Induced Pluripotent Stem Cells

**DOI:** 10.3389/fcell.2021.672039

**Published:** 2021-05-17

**Authors:** Xue Jiang, Yihuan Chen, Xiaofeng Liu, Lingqun Ye, Miao Yu, Zhenya Shen, Wei Lei, Shijun Hu

**Affiliations:** ^1^Department of Cardiovascular Surgery of The First Affiliated Hospital & Institute for Cardiovascular Science, Collaborative Innovation Center of Hematology, State Key Laboratory of Radiation Medicine and Protection, Medical College, Soochow University, Suzhou, China; ^2^The Affiliated Haian Hospital of Nantong University, Nantong, China

**Keywords:** induced pluripotent stem cell, inherited cardiomyopathy, disease modeling, heart disease, pathogenic mechanism

## Abstract

In the past decades, researchers discovered the contribution of genetic defects to the pathogenesis of primary cardiomyopathy and tried to explain the pathogenesis of these diseases by establishing a variety of disease models. Although human heart tissues and primary cardiomyocytes have advantages in modeling human heart diseases, they are difficult to obtain and culture *in vitro*. Defects developed in genetically modified animal models are notably different from human diseases at the molecular level. The advent of human induced pluripotent stem cells (hiPSCs) provides an unprecedented opportunity to further investigate the pathogenic mechanisms of inherited cardiomyopathies *in vitro* using patient-specific hiPSC-derived cardiomyocytes. In this review, we will make a summary of recent advances in *in vitro* inherited cardiomyopathy modeling using hiPSCs.

## Introduction

**FIGURE 1:**
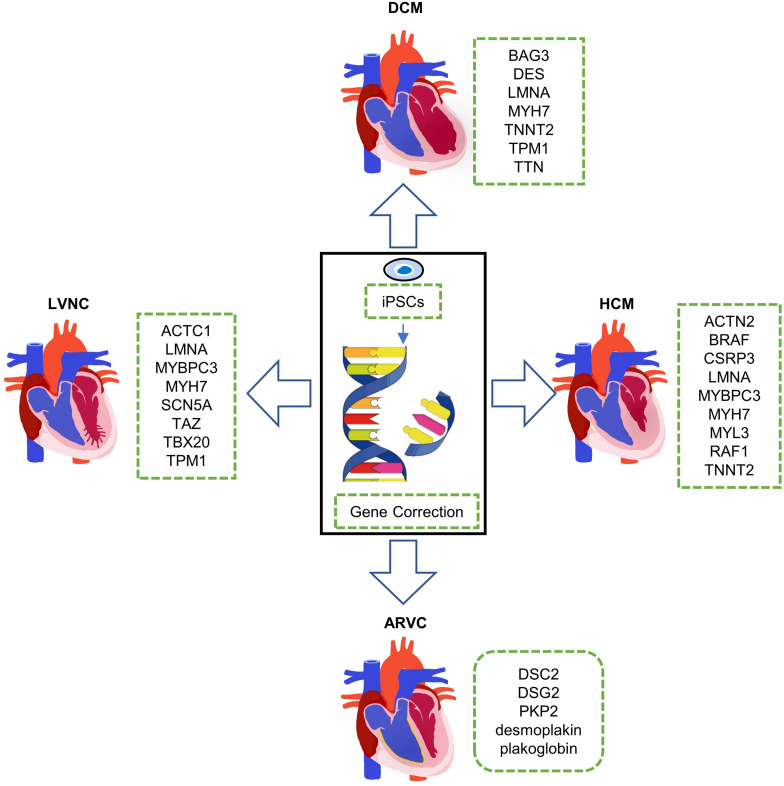
Clinical phenotypes of inherited cardiomyopathies and disease modeling with human induced pluripotent stem cells (hiPSCs). DCM, dilated cardiomyopathy; HCM, hypertrophic cardiomyopathy; ARVC, arrhythmogenic right ventricular; and LVNC, left ventricular non-compaction.

Inherited cardiomyopathy is a kind of myocardial disease with a range of different genetic disorders and epigenetic variations (Fatkin and Graham, 2002). Inherited cardiomyopathies mainly include dilated cardiomyopathy (DCM), hypertrophic cardiomyopathy (HCM), left ventricular non-compaction (LVNC), and arrhythmogenic right ventricular (ARVC) (Figure 1). Among these inherited cardiomyopathies, DCM is characterized by ventricular chamber dilatation and systolic dysfunction. It is usually related to mutations in genes such as *BAG3, LMNA, MYH7, TNNT2, TTN*, and so on. HCM is a disease with abnormal myocardial thickening. The cause of the disease is often closely related to the mutations of *ACTN2, BRAF, CSRP3, LMNA, MYL3*, and *TNNT2*, etc. Thickened myocardium makes it harder for the heart to pump blood. LVNC is characterized by the phenotype that the various tissue layers of the heart are not completely gathered together, resulting in a deep recess in the muscle wall. Patients with LVNC usually have symptoms of heart failure, arrhythmias, and thromboembolism and always accompanied by mutations in genes such as *ACTC1, MYBPC3, MYH7, SCN5A, TAZ, TBX20*, and so on. ARVC is a heart disease in which the right ventricular muscle is replaced by fat and/or scar tissue. With the aggravation of the degree, the right ventricle (RV) gradually loses its ability to pump blood, and it is mainly caused by mutations in genes such as *DSC2, PKP2*, and desmoplakin, etc. Patients with ARVC usually have arrhythmias and increased risk of cardiac arrest or death.

To reveal the pathogenesis of these genetic defects, a variety of disease models have been established. Human heart tissues and primary cardiomyocytes are the optimum choice undoubtedly, but they are difficult to obtain and culture *in vitro*, which hinders their application in cardiovascular research (Savla et al., 2014). Researchers then developed genetically modified animal models, among which mouse model is widely used. These animal models are crucial in progressing knowledge of the pathophysiology of heart disease because the basic principles of cardiac excitation and contraction are relatively conservative in the species. However, although animal models provide some useful results for the pathogenesis of cardiomyopathy, animals and humans are different at the molecular level, such as genetic background, epigenetic inheritance, transcriptional regulation, and protein modification. These, in turn, lead to significant differences between the functional and electrophysiological properties of the animal and human hearts, which limits the interpretation and applicability of these data (Ma et al., 2013). Therefore, the lack of a good source of live human cardiomyocytes *in vitro* and the failure to establish variant patient-specific disease models have significantly stymied research on inherited cardiomyopathy. Fortunately, human induced pluripotent stem cells (hiPSCs) provided a promising platform for genetic disease modeling.

Human induced pluripotent stem cells can be reprogrammed from somatic cells, which are easily isolated from the skin, urine, blood, and more unexpected samples, by virus-dependent or independent overexpression of a set of reprogramming factors such as Oct3/4, Sox2, Klf4, and c-Myc (Takahashi and Yamanaka, 2006). The hiPSCs can be indefinitely maintained and expanded in the pluripotent state *in vitro* and theoretically have the ability to generate any adult cell types (Kamp and Lyons, 2009). Through modulating BMP4 and WNT that are critical in heart development, hiPSCs are able to efficiently differentiate into cardiomyocytes (Qyang et al., 2007; Cao et al., 2013). Two major strategies have been developed for hiPSC-based inherited cardiomyopathy modeling: (1) reprogramming the somatic cells from genetic disease patients into hiPSCs and then differentiating them into cardiomyocytes and (2) performing gene editing to introduce pathogenic mutations into hiPSCs of healthy donor and then differentiating them into cardiomyocytes. Three precise genome-editing techniques, including zinc finger nuclease (ZFN; Kim et al., 2010), transcription activator-like effector nuclease (TALEN; Schmid-Burgk et al., 2013), and clustered regularly interspaced short palindromic repeat-associated protein 9 (CRISPR-Cas9; Wu et al., 2014), can be used to correct or introduce mutations in hiPSCs.

In this review, we will discuss the most common inherited cardiomyopathies abovementioned, including their genetic background and the work researchers have done so far using hiPSCs for mechanism research and symptom relief (Figure 1).

## Dilated Cardiomyopathy

Dilated cardiomyopathy is a common form of cardiomyopathy, which is characterized by ventricular chamber dilatation and systolic dysfunction (Maron et al., 2006). DCM contributes to hypertension, sudden cardiac death, progressive heart failure, and eventually leads to heart transplant (Wu et al., 2015). Recent studies have shown that cardiomyocytes from DCM patients often exhibit abnormal sarcomeric structure, which is likely due to pathogenic mutations in sarcomere protein-coding genes such as giant sarcomere protein titin (*TTN*), cardiac troponin T (*TNNT2*), tropomyosin 1 (*TPM1*), and desmin (*DES*; Fürst et al., 1988; Sehnert et al., 2002; Deacon et al., 2019). In recent years, the pathogenic genes of DCM and their pathological mechanism have been studied by establishing hiPSC-based cell models of DCM (Deacon et al., 2019).

Bearing significant roles in cardiac muscle, single TTN proteins extend from the *Z*-disk to the *M*-line, spanning the entire half-sarcomere length, and interact with the thick and thin filaments (Fürst et al., 1988). *TTN* truncating variants (TTNtv) are a major cause of genetic DCM, accounting for approximately 25% of familial cases (Herman et al., 2012). Hinson and his colleagues (Hinson et al., 2015) found that the TTNtv of DCM patients with TTN mutations in clinical practice are markedly enriched in the A-band. In order to understand the mechanism behind this phenotype, they generated patient-specific hiPSC-derived cardiomyocytes (hiPSC-CMs) with different *TTN* mutations, including two A-band TTNtvs (p.A22352fs or p.P22582fs) and one missense mutation (p.W976R) within the Z/I junction of *TTN* protein, as well as mutant isogenic hiPSC-CMs carrying CRISPR/Cas9-mediated TTNtvs in the I- or A-band exons. They observed considerable contractile dysfunction in both hiPSC-CM microtissues with I- or A-band TTNtvs, and attributed it to sarcomere insufficiency and aberrant responses to mechanical and adrenergic stress. However, previous studies revealed a tendency for TTN truncations in DCM to distribute to the A-band, while I-band TTNtvs are better tolerated and have been observed in healthy individuals (Herman et al., 2012; Roberts et al., 2015). Basing on RNA splicing analyses, Hinson and his colleagues further observed extraordinarily high rates of I-band exon incorporation into TTN transcripts in hiPSC-CMs (88% of TTN transcripts), five times more than that in adult left ventricle tissue (18% of TTN transcripts; Hinson et al., 2015). Thus, alternative RNA splicing-mediated elimination of I-band exon in adult cardiomyocytes might be a major mechanism for low incidence of I-band TTNtvs in DCM patients.

Cardiac troponin T, encoded by *TNNT2*, is another cardiac-specific sarcomere protein that involves in sarcomere assembly and cardiomyocyte contraction. Mutations of *TNNT2* could cause abnormal calcium ion dynamics and sarcomeric α-actinin, decreased contractility, and affect β-adrenergic signaling of cardiomyocytes. *TNNT2* mutation is also the leading cause of heart failure and sudden death in young athletes (Sehnert et al., 2002). By using patient-specific hiPSC model, Sun et al. (2012) demonstrated that hiPSC-CMs carrying *TNNT2* p.R173W mutation could partially recapitulate the disease phenotype of DCM, as indicated in altered regulation of calcium ion (Ca^2+^), decreased contractility, and abnormal distribution of sarcomeric α-actinin. Interestingly, metoprolol, a β1-selective β-blocker showing beneficial effect on the clinical symptoms of DCM patients, could improve the sarcomeric organization of DCM hiPSC-CMs (Sun et al., 2012). On this basis, Wu et al. found that p.R173W mutation could promote the nuclear localization of TNNT2, and alter the epigenetic regulation of key β-adrenergic signaling genes in DCM hiPSC-CMs (Wu et al., 2015). These studies provide novel targets for the treatment of DCM caused by *TNNT2* p.R173W mutation.

Bcl2-associated athanogene 3 (*BAG3*) encodes an anti-apoptotic co-chaperone protein, which is most highly expressed in skeletal and cardiac muscle and localizing to the sarcomeric *Z*-disk (Homma et al., 2006). Mutations in *BAG3* have been associated with DCM and muscular dystrophy (McDermott-Roe et al., 2019). However, heterozygous *BAG3* knockout mice failed to recapitulate the clinical phenotypes of heterozygous *BAG3* mutation-related cardiac disease (Homma et al., 2006). Two groups have explored the effects of DCM-related *BAG3* deficiency in cardiomyocytes, by using genome-editing approach to create both heterozygous and homozygous mutations in *BAG3* gene (Judge et al., 2017; McDermott-Roe et al., 2019). Despite the inconsistent observations at baseline, both groups demonstrated more serious fiber disarray in *BAG3*-deficient hiPSC-CMs under proteasome inhibition-induced proteotoxic stress. Consistently, nonsense mutations in BAG3 locus have been shown to cause BAG3 haploinsufficiency and disengagement of the BAG3-coordinated cardioprotective chaperone complex in hiPSC-CMs (Judge et al., 2017), which might be the pathologic mechanism of DCM-associated *BAG3* mutation. McDermott-Roe et al. demonstrated that stimulating the HSF1 (heat shock factor 1)-driven stress response pathway can reduce fiber disorder mediated by proteasome inhibition in hiPSC-CMs with *BAG3* p.R477H mutation (McDermott-Roe et al., 2019). This may help clinical treatment of DCM patients carrying *BAG3* mutations.

Some other sacromeric genes have also been involved in DCM. Deacon et al. (2019), through the establishment of hiPSC-CMs containing both sarcomeric gene *TPM1* mutation (p.E33K) and the costameric gene *vinculin* mutation (p.N220fs), demonstrated that composite genetic variants can combine and interact to induce DCM, especially when suffering from other disease-causing stressors. An unfamiliar heterozygous mutation of the desmin (*DES*), A285V mutation, was recently identified in DCM by Tse and colleagues through whole-exome sequencing (WES; Tse et al., 2013). They found that this mutation could cause three-dimensional structure changes of DES and then induce abnormal *DES* aggregations in hiPSC-CMs. Moreover, the mutation in β-myosin heavy chain 7 (*MYH7*) gene, which encodes myosin heavy chain (β-MHC), can also induce DCM. Yang et al. (2018) established an hiPSC-CMs line with *MYH7* mutation (p.E848G), and demonstrated that the reduced contractile function of single cells and engineered heart tissues with *MYH7* mutation (p.E848G) is due to the damaged interaction between *MYH7* and cardiac myosin-binding protein C.

In addition to mutations in sarcomere-related genes, mutations in nuclear membrane proteins can also cause DCM. The lamin A/C gene (*LMNA*) encodes two major nuclear lamina proteins lamins A and C through alternative splicing. Many *LMNA* mutations, including p.R225X, p.S143P, and p.K117fs, have been identified in familial DCM patients (Siu et al., 2012; Lee et al., 2019; Shah et al., 2019). The most common features of hiPSC-CMs derived from *LMNA* mutant DCM patients are arrhythmia, abnormal calcium handling, and increased sensitivity to electric and hypoxia stimulation (Shah et al., 2019). Many reports suggest that *LMNA* mutation-caused cardiac pathology is related to the activation of the PDGF pathway, and the symptoms caused by *LMNA* mutations can be moderated by the platelet-derived growth factor reactor β (PDGFRB) inhibitors and statins (Siu et al., 2012; Lee et al., 2019; Shah et al., 2019). Siu et al. (2012) demonstrated that under electrical stimulation, hiPSC-CMs carrying R225X or *LMNA* frameshift mutation present nuclear bleb formation and micronucleation. In the meanwhile, they found that the pro-apoptotic effects of field electric stimulation could be markedly attenuated by pharmacological blockade of mitogen-activated protein kinase/extracellular signal-regulated kinase (MAPK/ERK) pathway with U0126 and selumetinib (AZD6244; Siu et al., 2012).

Consistent with clinical disease manifestations, hiPSC-CMs with DCM mutations often exhibit phenotypes such as altered regulation of calcium ion and abnormal myocardial sarcomere structure. The advantage of modeling diseases through hiPSCs is that they cannot only summarize the disease phenotype but also its pathogenesis, which will greatly promote and deepen our understanding of the underlying mechanisms of DCM.

## Hypertrophic Cardiomyopathy

Hypertrophic cardiomyopathy is a disease of the cardiac sarcomere and tend to link to arrhythmia, electrophysiological abnormalities, and sudden cardiac death (Ramachandra et al., 2021). Practically, about 50% of the cases are caused by mutations either in *MYH7*, encoding β-myosin heavy chain, or in *MYBPC3*, encoding cardiac myosin-binding protein C (cMyBP-C; Birket et al., 2015), both of which are sarcomeric protein-encoding genes. Recently, researchers have also used hiPSCs to conduct HCM research (Prajapati et al., 2018; Tang et al., 2019; Ramachandra et al., 2021).

The *MYH7* is an important part of myosin and is related to contractile capacity of cells (Fontaine et al., 2021; Li et al., 2021). Studies have found that hiPSC-CMs carrying a missense mutation (p.R663H) in *MYH7* displayed the phenotype of cellular enlargement, contractile arrhythmia at the single-cell level, and dysregulation of Ca^2+^ cycling and elevation in intracellular Ca^2+^ ([Ca^2+^]i). The main cause of these defects is the continuous increase in [Ca^2+^]i, which leads to the activation of calcineurin that further dephosphorylates NFAT3 transcription factors, allowing them to translocate to the nucleus and interact directly with classical transcript factors such as GATA4 and MEF2 (Lan et al., 2013). In hiPSC-CMs carrying p.R442G mutation in *MYH7*, Han et al. (2014) observed sarcomere disorders, indicating the reduction of contractility in HCM hiPSC-CMs and an abnormal Ca^2+^ handling. Treatment of verapamil, a calcium channel blocker, successfully alleviated calcium handling abnormalities and arrhythmia in HCM hiPSC-CMs with *MYH7* p.R663H or p.R442G mutations (Lan et al., 2013; Han et al., 2014). In addition, some Na^+^ channel blocker such as lidocaine, mexiletine, and ranolazine, but not drugs targeting K^+^ channels, are also able to restore normal beat frequency in HCM hiPSC-CMs (Lan et al., 2013). These evidences provide some theoretical basis for the treatment of HCM caused by *MYH7* mutation.

The mutation of *MYBPC3*, encoding a thick filament contractile protein that regulates sarcomere organization and cardiac contractility, is another important cause of HCM, which in turn causes damage to the contractile ability of cardiomyocytes and ultimately leads to HCM (Jin et al., 2020). Birket et al. (2015) found that the amount of MyBPC3 protein was reduced by nearly half in *MYBPC3*-mutant (c.2373dupG) hiPSC-CMs when compared with the normal level. Due to the haploinsufficiency, the maximum Ca^2+^-activated force markedly decreased. Premature termination codon (PTC) is another common mutation form of *MYBPC3* that causes HCM. Seeger et al. found that PTC mutation led to nonsense-mediated decay (NMD) and ubiquitin–proteasome system (UPS), leading to haploinsufficiency of healthy alleles and ultimately to HCM. Inhibition of the NMD pathway in HCM hiPSC-CMs carrying *MYBPC3* (p.R943X) mutation resulted in the restoration of molecular phenotypes and calcium-processing kinetics (Seeger et al., 2019). These studies have shown that when dealing with HCM caused by mutations in the *MYBPC3* gene, targeting the NMD pathway might be effective.

In addition to sarcomeric genes, mutations in some genes related to cell signaling pathways can also cause HCM. The BRAF protein is a RAF family serine/threonine protein kinase involved in RAS/MAPK signaling. Researchers have found that its mutations can cause pro-fibrotic phenotype, enhanced susceptibility to arrhythmias, aberrant MEK1/2 signaling and sarcomere disorders, and eventually lead to HCM (Cashman et al., 2016; Josowitz et al., 2016; Jaffré et al., 2019). Josowitz et al. used hiPSC-CMs to construct disease models carrying *BRAF* p.T599R or p.Q257R mutations. They sorted out SIRPα^+/^CD90^–^ and SIRPα^–^/CD90^+^ cardiomyocytes so as to examine hiPSC-derived cell type-specific phenotypes and cellular interactions underpinning HCM. They found that *BRAF*-mutant SIRPα^+^/CD90^–^ cardiomyocytes displayed the phenotype of HCM, and *BRAF*-mutant SIRPα^–^/CD90^+^ cells exhibited a pro-fibrotic phenotype and partially modulated cardiomyocyte hypertrophy through transforming growth factor (TGF) paracrine signaling. These symptoms can be relieved by inhibition of TGFβ or RAS/MAPK signaling (Josowitz et al., 2016). Furthermore, hiPSCs from a patient with cardio-facio-cutaneous syndrome were also used to develop the 3D human-engineered cardiac tissue model of *BRAF* p.T599R mutation-induced HCM. This model reflects an enhanced susceptibility to arrhythmias (Cashman et al., 2016). The *RAF1* p.S257L mutation also demonstrated potential to cause cell-autonomous hypertrophy and myofibrillar disarray in hiPSC-CMs, through activation of ERK5 and MEK1/2 signaling, respectively, (Jaffré et al., 2019).

Moreover, researchers have also established disease models through hiPSCs to study HCM caused by mutations in genes such as SCO cytochrome oxidase-deficient homolog 2 (*SCO2*), *CSRP3*, which encodes muscle LIM protein, and *ACTN2*, encoding α-actinin 2 (Hallas et al., 2018; Li et al., 2019; Prondzynski et al., 2019), which are also some common genetic mutations that cause HCM. These mutations can, respectively, result in reduced mitochondrial oxidative ATP production capacity, multinucleation, disorganized sarcomeric ultrastructure, and higher myofilament Ca^2+^ sensitivity (Hallas et al., 2018; Li et al., 2019; Prondzynski et al., 2019).

Diastolic dysfunction (DD) is commonly diagnosed in HCM patients, and is a major cause of morbidity and mortality among HCM patients. Wu et al. (2019) generated four hiPSC-CM models of DD that carry different mutations in sarcomeric proteins, including *MYH7* (p.R663H), *MYBPC3* (p.V321M), *MYBPC3* (p.V219L), and *TNNT2* (p.R92W). These human hiPSC-CM models recapitulated the disease phenotype of DD in HCM, as evidenced by shortened diastolic sarcomere length, decreased maximum relaxation rate, and prolonged relaxation duration. Both diastolic Ca^2+^ overload and enhanced myofilament Ca^2+^ sensitivity are involved in impaired diastolic function in HCM hiPSC-CMs, which can be alleviated by treatment with partial Ca^2+^ or late Na^+^ current inhibitors (Wu et al., 2019).

On the one hand, the above research shows that hiPSC-CMs with HCM pathogenic genes often have the characteristics of cellular enlargement, contractile arrhythmia, and disorganized sarcomeric, which are consistent with their arrhythmia and electrophysiological abnormalities in diseased organs. On the other, these studies point out the direction for further exploring the possible pathogenesis of HCM and provide a theoretical basis for the rational formulation of clinical protocols. The hiPSC-CM-based HCM studies have shown that abnormal calcium homeostasis is an important factor leading to inherited HCM. The development of drugs that inhibit Ca^2+^ and Na^+^ channels may provide a direction for HCM treatment.

## Left Ventricular Non-Compaction

Left ventricular non-compaction is caused by an arrest of normal endomyocardial morphogenesis. Patients with LVNC often have symptoms of heart failure, arrhythmias, and thromboembolism. LVNC is mainly related to mutations in genes encoding taffazin (*TAZ*), lamin A/C (*LMNA*), sodium channel type Vα (*SCN5A*), *MYH7*, *MYBPC3*, α-cardiac actin 1 (*ACTC1*), and α-tropomyosin (*TPM1*). Among them, researchers have established hiPSC-CMs models of *ACTC1*, *TPM1*, and *TBX20* genes with mutations to study the pathological mechanisms of LVNC.

Among them, *ACTC1* and *TPM1* are genes encoding sarcomere proteins. To elucidate the pathological role, hiPSCs from a patient carrying a E99K-ACTC1 (α-cardiac actin 1) mutation has been developed and studied (Smith et al., 2018). Smith et al., through the study of two different training methods, 3D and 2D, found that E99K-ACTC1 hiPSC-CMs have obviously arrhythmogenesis in both kinds of models, that is because of the abnormal Ca^2+^ handling, such as prolonged Ca^2+^ transients (Smith et al., 2018). Takasaki et al. found that hiPSC-CMs carrying a p.R178H mutation in *TPM1* exhibited obvious pathological changes, such as disturbed sarcomere structure and impaired calcium handling in cardiomyocytes. Microarray analysis showed that *TPM1* mutations lead to down-regulated expression of many genes involved in heart development and beneficial regulation cellular processes, particularly the calcium signaling pathway (Takasaki et al., 2018).

In addition, researchers have also studied many other mutations associated with LVNC by using patient-specific hiPSC-CMs. LVNC hiPSC-CMs carrying p.T262M mutation and stop-gain mutation p.Y317^∗^ in *TBX20* (T-box transcription factor 20) possess a defect of proliferative capacity due to abnormal activation of TGF-β signaling. Compared with mild DCM hiPSC-CMs, LVNC hiPSC-CMs have a significantly higher expression of TGF-β signaling pathway-related genes, while the expression of PRDM16, the repressor of TGF-β signaling, was significantly down-regulated in all LVNC hiPSC-CMs. These data indicate that the abnormal activation of TGF-β signaling by *TBX20* mutation is associated with the proliferation defect of LVNC hiPSC-CMs (Kodo et al., 2016). These studies provide more possibilities for the follow-up investigators’ work and lay the foundation for the clinical treatment of LVNC.

## Arrhythmogenic Right Ventricular

Arrhythmogenic right ventricular is an inherited primary heart muscle disorder characterized by fibrofatty infiltration of the myocardium and cardiomyocyte loss basically in the RV that may lead to sudden cardiac death (Chen et al., 2020; Jahng et al., 2021). More than 50% of the ARVC patients had mutations in the desmosome genes including plakophilin 2 (*PKP2*), desmoglein 2 (*DSG2*), desmoplakin, and plakoglobin (Lombardi and Marian, 2010).

Among them, the most common mutations occur in the *PKP2* gene (Calkins and Marcus, 2008), which is important for desmosomal protein localization, lipid formation, β-catenin activity, and the activity of Wnt signaling pathway. For this reason, in recent years, researchers have begun to use hiPSCs to construct disease models of ARVC caused by *PKP2* mutations. In the beginning, Ma and his colleagues investigated the role of 1841T > C mutation of *PKP2* gene in hiPSC-CMs. The *PKP2* mutant hiPSC-CMs exhibit reduced localization of desmosomal proteins on the cell surface, enlarged cardiomyocytes, and a more lipid formation. They also found that after exposure to adipogenic differentiation medium for 2 weeks, the lipid content of ARVC-hiPSC-derived cardiomyocytes was significantly higher than that in the control group (Ma et al., 2013). In hiPSC-CMs with the c.2484C > T mutation of *PKP2*, Kim and his colleagues manifested abnormal nuclear translocation of plakoglobin and decreased β-catenin activity in cardiogenic conditions (Kim et al., 2013). However, these hiPSC-CMs cannot reproduce the pathological phenotypes of ARVC in standard cardiogenic conditions only if adjusting the cultivation conditions to mature them (Kim et al., 2013). Khudiakov et al. (2015) using hiPSC-CMs with two mutations (c.23321del.T, p.K859R) in the *PKP2* gene, found that the *PKP2* mutations could decrease the activity of Wnt signaling pathway during adipogenic and cardiomyogenic differentiation. To improve this hiPSC-CMs model, Wen et al. developed a method to induce metabolic maturation of hiPSC-CMs. Moreover, they found that coactivation of normal peroxisome proliferator-activated receptor (PPAR)-α and abnormal PPAR-γ pathways could stimulate the pathological signatures such as excessive CM adipogenesis, CM apoptosis, Na^+^ channel downregulation, and intracellular calcium deficiency of ARVC in hiPSC-CMs carrying two mutations (c.2484C > T, c.2013delC) in *PKP2* (Wen et al., 2015). The abovementioned studies indicate that induction of adult-like metabolic energetics is essential to model a cardiac disease with patient-specific hiPSCs.

Besides, Padrón-Barthe et al. demonstrated the genetic mechanism of ARVC5, the most aggressive ARVC subtype, by using the hiPSC-CM model. ARVC5 is a devastating disease that is caused by a point mutation in *TMEM43*, a transmembrane protein that is located in the nuclear membrane. Their research demonstrated that the hiPSC-CMs with p.S358L mutation in *TMEM43* had an increased contraction duration, time to peak, and relaxation time in mutants, and these contraction abnormalities could be relieved by inhibition of glucogen synthase kinase 3 β (Padrón-Barthe et al., 2019). In addition to mutations in desmosomal and nuclear membrane proteins that can cause ARVC, abnormalities in ion channels can also cause ARVC. Cardiomyocytes derived from *DSG2* mutant hiPSCs displayed multiple ion channel dysfunction, cellular electrophysiological abnormalities, and increased sensitivity to adrenergic stimulation, which may underlie ARVC patients’ arrhythmias (El-Battrawy et al., 2018). In the above studies, it was found that hiPSC-CMs derived from patient’s somatic cells showed various phenotypes such as enlarged cardiomyocytes, increased lipid formation, and abnormal cell contraction, which are related to the fibrofatty infiltration of the myocardium in ARVC patients. Therefore, patient-specific hiPSC-CMs have been shown to be beneficial in studying the pathogenesis of ARVC.

In addition to the abovementioned cardiomyopathies, researchers have also used iPSCs to study restrictive cardiomyopathy (RCM), a rare heart disease, which is usually caused by increased myocardial stiffness, which results in limited ventricular filling (Galea et al., 2020). Brodehl et al. constructed a disease model with p.Y122H mutation of *DSE* by using iPSCs and found that the mutant desmin with a severe filament assembly defect supports the pathogenicity of the RCM. By establishing a disease model of this mutation, they first classified the p.Y122H mutation of *DSE* as a novel pathogenic mutation (Brodehl et al., 2019).

## Outlook

**FIGURE 2:**
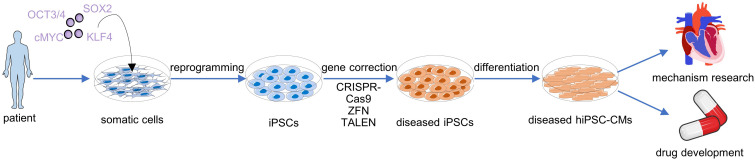
The whole process of constructing hiPSC-derived cardiomyocyte (hiPSC-CM) disease model from patients’ somatic cells and the application of these disease models.

As one of the increasingly serious problems of the 21st century, cardiomyopathy still has many difficulties waiting for us to overcome, for example, the pathogenesis of many genetic diseases has not yet been clarified, and the corresponding treatment options have not been studied. As we described above, hiPSC technology is a powerful means that can be used (Figure 2). Through this technology, researchers have established various disease models of cardiomyopathy, such as HCM, DCM, ARVC, and LVNC, and studied the mechanisms of pathogenesis caused by mutations of different genes. Therefore, hiPSC-based cardiovascular disease models provide more possibilities for the follow-up investigators’ work and will greatly promote and deepen our understanding of the underlying mechanisms of cardiomyopathy. In addition to these 2D disease models, researchers have also constructed 3D organoid models to simulate real human organs and relieve the limitations of spatial factors on disease models (Giacomelli et al., 2020). In addition, iPSC-CMs is also widely used in drug screening (Ito et al., 2020; Li et al., 2020), and iPSC-CM and its derivatives are also used to treat heart failure (Nakamura and Murry, 2019). These all lay the foundation for the clinical treatment of cardiac disease.

However, at present, hiPSC-CMs generally have the problem of immaturity. Mature and immature cells have huge differences in their cell morphology, number of mitochondria, sarcomere structure, calcium transient, and electrophysiology, etc. In addition, the maturation of cardiomyocytes will bring more detailed subtypes, such as ventricular myocardium, atrial myocardium, and sinoatrial node cells, etc. All of these have a significant impact on our research on specific diseases. If the subtypes of differentiated and mature cardiomyocytes can be obtained, we can make more detailed and precise studies on the physiological processes of diseases. Besides, the cellular and molecular crosstalk between cardiac cell lineages is difficult to model by using only hiPSC-derived cardiomyocytes in a two-dimensional model. Therefore, we should use hiPSCs to differentiate a variety of cardiac cells, such as endothelial cells, and construct a three-dimensional model of the cardiac cells to better simulate the overall changes in the heart and the interaction between cells when the disease occurs. In summary, hiPSC technology will not only completely alter our understanding of diseases but also will eventually change the future of medical practice and make personalized medicine possible.

## Author Contributions

XJ, YC, and XL mainly wrote the manuscript. LY, MY, and ZS revised the manuscript. WL and SH wrote and revised the manuscript, as well as provided the funding. All authors contributed to the article and approved the submitted version.

## Conflict of Interest

The authors declare that the research was conducted in the absence of any commercial or financial relationships that could be construed as a potential conflict of interest.
